# RF Energy Harvesting IoT System for Museum Ambience Control with Deep Learning

**DOI:** 10.3390/s19204465

**Published:** 2019-10-15

**Authors:** Nermeen A. Eltresy, Osama M. Dardeer, Awab Al-Habal, Esraa Elhariri, Ali H. Hassan, Ahmed Khattab, Dalia N. Elsheakh, Shereen A. Taie, Hassan Mostafa, Hala A. Elsadek, Esmat A. Abdallah

**Affiliations:** 1 Electronics Research Institute (ERI), Giza 12622, Egypt; nermeen@eri.sci.eg (N.A.E.); osamadardeer@eri.sci.eg (O.M.D.); daliaelsheakh@gmail.com (D.N.E.); helsadek@mcit.gov.eg (H.A.E.); esmataa2@hotmail.com (E.A.A.); 2 Electronics and Electrical Communication Engineering Department, Cairo University, Giza 12613, Egypt; awab.habal.95@gmail.com (A.A.-H.); ali.elhussien@gmail.com (A.H.H.); hmostafa@zewailcity.edu.eg (H.M.); 3 Computer Science Department, Faculty of Computers and Information, Fayoum University, Fayoum 63514, Egypt; emh00@fayoum.edu.eg (E.E.); sat00@fayoum.edu.eg (S.A.T.); 4 University of Science and Technology, Nanotechnology and Nanoelectronics Program, Zewail City of Science and Technology, 6th of October City 12578, Egypt

**Keywords:** ambience monitoring, antenna array, deep learning, Internet of Things (IoT), rectenna, RF energy harvesting, time series prediction

## Abstract

Museum contents are vulnerable to bad ambience conditions and human vandalization. Preserving the contents of museums is a duty towards humanity. In this paper, we develop an Internet of Things (IoT)-based system for museum monitoring and control. The developed system does not only autonomously set the museum ambience to levels that preserve the health of the artifacts and provide alarms upon intended or unintended vandalization attempts, but also allows for remote ambience control through authorized Internet-enabled devices. A key differentiating aspect of the proposed system is the use of always-on and power-hungry sensors for comprehensive and precise museum monitoring, while being powered by harvesting the Radio Frequency (RF) energy freely available within the museum. This contrasts with technologies proposed in the literature, which use RF energy harvesting to power simple IoT sensing devices. We use rectenna arrays that collect RF energy and convert it to electric power to prolong the lifetime of the sensor nodes. Another important feature of the proposed system is the use of deep learning to find daily trends in the collected environment data. Accordingly, the museum ambience is further optimized, and the system becomes more resilient to faults in the sensed data.

## 1. Introduction and Related Work

### 1.1. Introduction

Museums are not just places to conserve human cultural heritage, but also critical tools for history and arts. Preserving a museum’s contents from potential damages caused by natural degeneracy, inappropriate environmental conditions, or human vandalization is a duty towards present and future generations. This necessitates the precise monitoring and control of the museum’s microclimate, pollution, and lightening, such that its content is exposed to environmental conditions that cause the least harm and reduce the natural degeneracy rate. Traditionally, psychrometers, hygrometers, and other conventional measurement equipment were used to measure museum ambience parameters. However, such tools have large sizes that need human intervention and are unable to provide continuous measurements [1]. 

Alternatively, the Internet of Things (IoT) paradigm provides the ultimate solution for museum ambience control [2]. An IoT system is typically composed of a Wireless Sensor Network (WSN) that collects the needed data and relays it to the Internet for further processing, storage, and decision-making. Thus, an IoT-based museum ambience control will enable preservation of the human cultural heritage at low cost and high flexibility with insignificant visual impact on the artifacts. Furthermore, it allows for continuous and remote monitoring and control of the museum ambience. 

One of the major challenges that WSN and IoT systems face is the limited lifetime of the battery-powered nodes. While the development of energy-efficient nodes has been widely investigated, energy harvesting has recently presented itself as the key solution to significantly prolong the nodes’ lifetime. Energy from external sources, such as solar energy, thermal energy, mechanical energy, and radio frequency (RF) energy, can be harvested. RF energy harvesting is considered as one of the most durable power sources for different WSN and IoT applications. This is because the ambient RF energy to be harvested is available everywhere and at any time of the day, when compared to other energy harvesting sources such as solar energy [3,4]. RF energy harvesting can be performed at mobile applications frequency bands, such as GSM 900 MHz, GSM 1800 MHz, UMTS 2100 MHz, and LTE 2600 MHz. It can also be performed at Wi-Fi 2.4 GHz and 5 GHz. These bands typically contain significant amounts of energy that can be harvested. 

In this paper, we present an integrated IoT system for museum ambience control. The proposed system is powered using RF energy in a museum ambience and uses deep learning technique for improved control. The contributions of the paper are as follows:

- A complete energy harvesting solution using planar antenna arrays with a simple one-layer structure, integrated with a highly sensitive and simple structure wideband rectifier, which is a suitable solution for powering wireless sensor network an in IoT system. We use two antenna array designs for the RF energy harvesting system to obtain the maximum harvested energy, depending on the scenario in which the system is operating. The first design is a circularly polarized (CP) 2 × 2 antenna array. The second design is a dual linearly polarized antenna array (DLPAA). The complete design of these antennas and some of the other individual elements are presented in [5,6,7,8]. In this paper, we integrate each of the designed antenna arrays with the appropriate rectifier through suitable matching circuits to get maximum power transfer and alleviate the mismatch losses. Furthermore, a power management unit is designed to boost the collected RF energy to charge the battery.
- The development of a layered IoT architecture for museum ambience control based on four layers: (1) A physical interface layer that collects the ambience data and implements decisions developed to control the environment parameters; (2) a network connectivity layer that connects the physical interface layer to the network edge; (3) a fog layer that preprocesses the collected data and performs time-critical actions; and (4) a cloud layer at which the data is stored for further analysis. The building blocks of the architecture are designed for ultra-low-power operation such that it can exploit RF energy harvesting for a prolonged lifetime, despite the use of always-on sensors for occupancy detection and touch detection. This contrasts with existing platforms in the related literature that are battery-powered [2,9,10,11,12], and hence, have a limited lifetime and are incapable of using occupancy and touch-detection sensors.
- The development of a deep learning-based framework for indoor air quality prediction and control that predicts the ambience conditions in the upcoming 24 hours. The proposed framework makes the system robust and fault tolerant by providing another source of ambience conditions, such that if the sensors’ readings coming from the IoT system are disrupted for any reason, the museum control is not affected. Furthermore, predicting the future of the ambience helps in creating dynamic ambience control plans with lower energy consumption that provide the basis on which the IoT system operates. This framework enhances our preliminary design presented in [13] by: (1) adding new date-time features (week day, day hour) to consider the effects of working hours and working days, (2) performing correlation analysis to investigate the most suitable predictive variables that affect each other, and (3) using data augmentation to enhance deep learning performance and avoid overfitting. These enhancements significantly reduce errors in predicting environmental attributes by up to 50%, compared to [13].
- The implementation of a prototype that integrates the various components of the system. An extensive set of experiments is presented to demonstrate its performance.

### 1.2. Related Work

We briefly review the related research literature of the overall museum control system as well as its main components. Even though there exist several commercial ambient monitoring and control systems for environments such as offices, homes, and buildings, they are not discussed in this paper. Such commercial systems (1) focus on only a single element, such as sensor nodes or IoT web infrastructure; and (2) not directly applicable in museum environments. The latter is because every artifact or display must contain a sensor node to use the touch detection feature and have localized visitors’ information for every artifact. Furthermore, museums have different conditions for visitors. The number of visitors and the frequency and timing of visits differs from other environments (homes or residential buildings).

#### 1.2.1. Museum Ambience Monitoring and Control

Recently, many IoT-based applications have been adopted in museums. These applications make use of the Internet’s main characteristics (availability, scalability, …, etc.). There are three main applications of using IoT in a museum environment: museum ambience monitoring [2,9,10,11,12], smart museum [14,15,16,17], and museum surveillance [18,19]. 

Museum ambience monitoring has gained more attention compared to other applications because of the importance of preserving museum contents for a period of time. For instance, Wireless Sensor Network (WSN) is the main concept used in the literature. Efforts have been taken to design systems that contain only WSN, without including connectivity to the Internet, as in [10]. Other designs (such as [2]) exploit cloud connectivity to monitor ambient attributes from a mobile phone. In [11], a complete solution is proposed, which includes WSN with connectivity to cloud and control capabilities. Unlike most contributions in the literature [2,9,10,11,12], we propose a system that combines RF energy harvesting with an IoT-layered architecture and data analysis capabilities to monitor and control museum ambience.

#### 1.2.2. Radio Frequency (RF) Energy Harvesting and Antenna Design

In order to capture as much RF power as possible, antenna arrays are generally applied, instead of a single element. Hence, larger aperture size tends to collect more incoming RF energy. Different rectennas have been presented in the literature, which include an antenna array as the receiving unit to collect the RF energy [20,21,22]. Usually, the aperture-coupling feeding technique is used for feeding a single patch with crossed narrow slots or two offset narrow slots. A small-size wideband dual linear polarization array was presented in [23]. The array uses two elements of balanced antipodal Vivaldi antenna. The authors in [24] introduced a dual-polarized and dual-frequency antenna for radio frequency energy harvesting at GSM 900, and ISM 2.45 GHz. However, the antenna structure is complex because it consists of multilayers. A Trible band C/X/Ku (bands) dual polarized sub antenna array has 11 layers designed for specific apportion ratio application (SAR) [25]. Different designs have been reported [26,27,28] to present CP antenna arrays. All these antenna array structures are complex and not suitable for RF energy harvesting application because in the RF energy harvesting system the antenna must be integrated with a rectifying circuit and suitable matching unit. It is difficult and complex to integrate the rectifier and matching units in a multilayer and dual port structure.

#### 1.2.3. Deep Learning via Time Series Prediction 

Time series data prediction is challenging due to its noisy and unpredictable features [29]. The analysis of historical data collected from different sources helps predict events and forecast a time series data, which subsequently proposes strategies to reduce energy consumption of continuous data collection and transmission of such data [30]. However, time series prediction does not have data constraints, as in other techniques, such as sub-sampling [31]. Thus, several research works have tackled the problem of time series prediction (e.g., [32,33,34]). In [32] and [33], two different machine learning-based approaches for indoor temperature prediction were proposed. In [29], four models based on Support Vector Machine (SVM), considering a different combination of various factors, were established. While in [33], an artificial neural network-based model considering both indoor and outdoor parameters was proposed for energy saving. A two-stage methodology was developed to predict indoor temperature. Moreover, in [34], a deep learning-based single-step time series prediction model for indoor air quality was proposed. Both Long Short-Term Memory (LSTM) and Gated Recurrent Unit (GRU) models are utilized, along with an optimal time step searching algorithm, for indoor air quality prediction. The results show that the GRU model beats both LSTM and linear regression models.

## 2. Methodology

### 2.1. Overall System Description

**Figure 1:**
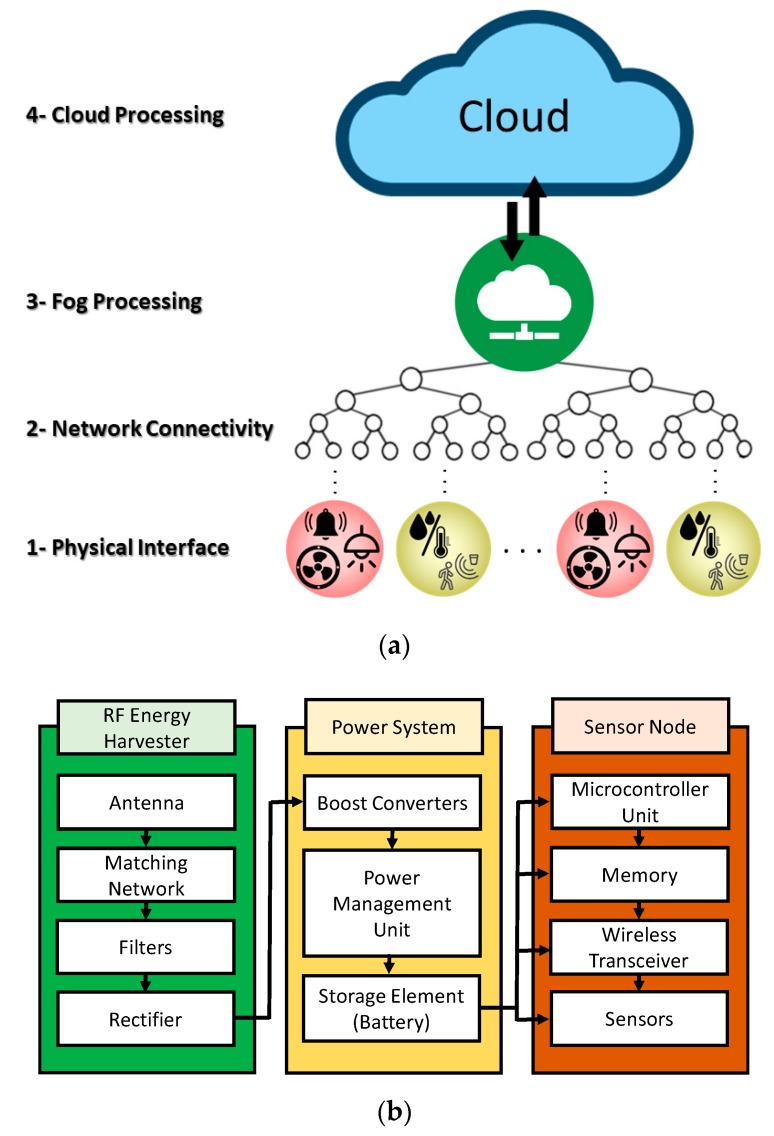
Illustration of the Internet of Things (IoT) system architecture and node design: (**a**) Proposed layered IoT architecture for museum control; (**b**) block diagram of the Radio Frequency (RF) energy harvesting sensor node.

The proposed ambience monitoring system allows the automation, remote control, and optimization of the artifact display environment inside museums. It relies on a set of sensor nodes that track the environment attributes that directly impact the display ambience, such as room occupancy, temperature, humidity, light intensity, and carbon monoxide (CO) and carbon dioxide (CO_2_) levels. The system accordingly adjusts the display room ambience by controlling the actuator nodes connected to the heating, ventilation, air conditioning (HAVC), room entrance and lights, etc. The collected data is passed to the Internet to exploit its advantages with ubiquitous connectivity and deep data analysis. Figure 1a illustrates the IoT layered architecture developed in this paper.

A unique feature of the proposed system that distinguishes it from prior works [2,9,10,11,12] is that the used sensor nodes are powered by harvesting the RF energy available in the museum. This does not only remove the bounds on the system’s lifetime, but also makes the system greener. Figure 1b depicts a block diagram of the developed RF energy-harvesting wireless sensor node. The node is composed of three systems: RF energy harvesting system, power system, and sensor node.

The RF energy harvesting system collects the ambient RF electromagnetic energy and converts it into electric power. This function is achieved through the design of a multi-band antenna that operates over the bands from which the RF energy is harvested. The designed antenna acts as a transducer. The antenna is followed by a matching circuit, filters, and a rectifier circuit to convert and improve the characteristics of the collected energy.

The power system uses the rectified power, boosts it, and stores it in a power storage element, such as a battery or a super capacitor. A boost converter first performs DC-to-DC conversion to amplify the harvested power. A power management unit (PMU) effectively optimizes the recharging process of the ‘main’ power sources, such as a battery, using the collected power. In our system, the main power source and power storage system are combined into a single element—a rechargeable battery that supplies the different components of the sensor node with needed power. 

It is worth noting that all other nodes in the system—the actuator nodes and the gateway—are connected to the mains power source. This is because such nodes are connected to the HVAC devices or access points, which are typically mains-powered. Hence, the sensor node is the only node that is powered through RF energy harvesting.

### 2.2. RF Energy Harvesting System Design

An RF energy harvester is typically composed of a receiving antenna, a band pass filter, a matching network, a rectifier, and a terminal load [35,36]. In this paper, we present and fabricate two of our RF energy harvesting systems designs, to integrate them with the proposed IoT museum control system. Studying two designs, namely CP and DLPAA, allows us to obtain maximum harvested energy, depending on the scenario in which the system is operating (this depends on the amount and direction of the ambient RF energy). While one design can be better in a certain setup, the other can offer more harvested energy in a different setup. The detailed designs of the antennas, the feeding network, and their parameters are available in [5,6,7,8].

#### 2.2.1. CP Array Design

**Figure 2:**
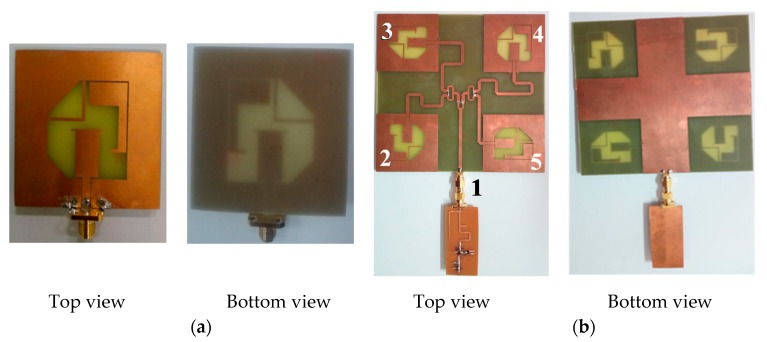
The fabricated CP rectennas: (**a**) Single antenna element, top and bottom layer; (**b**) 2 × 2 circularly polarized rectenna array, top and bottom layer.

The first design uses a CP 2 × 2 antenna array. The use of single elements with the conventional rectangular patch antenna and dependence on the feeding network to obtain circular polarization results in single narrow bandwidth. In contrast, our single element antenna (shown in Figure 2a) has many advantages, as it provides a dual circularly polarized wide bandwidth, and consequently provides more harvested energy (see [7] for more details). A sequential phase feeding network is required in order to constitute a 2 × 2 CP antenna array. The feeding network is a corporate microstrip feeding network that performs impedance matching and phase controlling functions. In order to achieve CP radiation, a sequential feeding technique was applied to both elemental orientation and phase distribution. The four elements are arranged in a 0°, 90°, 180°, and 270° manner to achieve symmetry and good CP operation. The spacing between the elements is 0.768λ_0_ at a frequency of 2.45 GHz. To achieve the required differential phases between the elements, the length of transmission lines to each antenna was increased by 0.25 times the guided wavelength to achieve a 90° increase in phase delay. Figure 2 illustrates the feeding network for the 2 × 2 array. A ground plane is placed below the microstrip substrate for the Wilkinson divider.

The complete 2 × 2 array was designed on FR4 substrate with a dielectric constant of 4.3, thickness of 1.6 mm, and loss tangent of 0.025. In order to connect the feeding system to the elements, a microstrip-line-to-CPW transition [37] was applied via holes. The feeding network was fed with an SMA panel connector attached at the right side of the board. The total size of the 2 × 2 array is 148 × 148 mm^2^.

A rectifier circuit was designed and implemented for the conversion process. The topology of the conversion circuit used in this paper is the voltage doubler full wave rectification circuit. The Schottky diode HSMS-2860 was selected as the rectifying device. Before the impedance matching network could be designed, the input impedance of the voltage doubler circuit had to be calculated. This process has been done using the Advanced Design System (ADS) simulator. The real and imaginary parts of the input impedance at 0 dBm were found to be 1.046–j102.68 Ω at 2.45 GHz, while at 5 GHz, the impedance was 1.142–j34.1 Ω.

According to [38], the П section model was used as a dual band impedance matching transformer for unequal complex impedance loads. The distance between the voltage doubler circuit and the matching network was implemented in the form of a meander line for size reduction. In addition, the tuning stub was printed as an L-shaped stub for miniaturization. The characteristic impedance *Z_c_* and electrical length *θ_c_* of the sections constituting the П-model structure were initially calculated according to [38]. Then, the corresponding dimensions were optimized using an ADS simulator. The rectifier was fabricated on FR4 substrate with a dielectric constant of 4.3, thickness of 0.8 mm, and loss tangent of 0.025. The total size of the rectifier was 64 × 28.8 × 0.8 mm^3^. Figure 4 shows the fabricated rectifier.

By combining the antenna array with the designed rectifier, a complete rectenna was formed. Figure 2 shows the fabricated rectenna. An adapter (SMA male-to-male) was used to connect the antenna array to the rectifier.

#### 2.2.2. DLPAA Design

**Figure 3:**
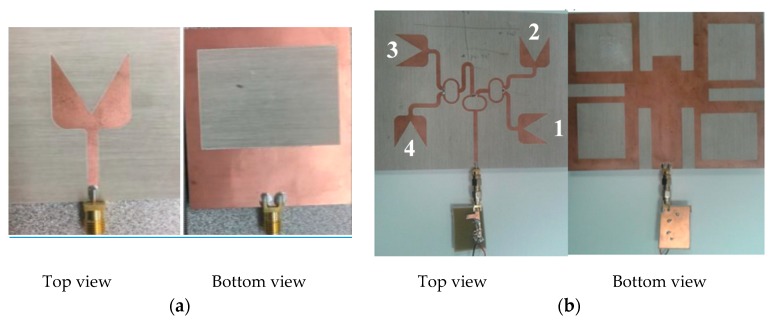
The fabricated DLPAA rectennas: (**a**) Single antenna element, top and bottom layer; (**b**) 2 × 2 dual polarized antenna array, top and bottom layer.

The second design uses a dual linearly polarized antenna array (DLPAA). The design of the DLPAA was planar in order for it to be integrated with the RF energy harvesting system. As a result, a 2 × 2 DLP antenna array, as shown in Figure 3b, was designed, based on the antenna element [5]. The antenna elements were oriented such that the antenna array supports DLP, as shown in Figure 3b. The four elements in the array were arranged to achieve a dual polarized radiation pattern. There is a 180° phase shift between element 1 and element 3, which makes them radiate vertically in the same direction. In addition, there is a 180° phase shift between element 2 and element 4 such that they radiate horizontally in the same direction. Thus, a dual polarized radiation pattern is achieved [5,8]. The design consists of a modified ground plane with a rectangular-shaped slot and a V-shaped planar monopole radiator. The modified ground plane has a rectangular shaped slot with dimensions of Ls × Ws = 30 × 40 mm^2^, used to improve the antenna matching and gain. The detailed design steps and advantages of the proposed antenna array are presented in [5], where the effect of each part is studied. The radiation mechanism for this antenna was completely different from the fully ground microstrip antenna, resulting in a wider impedance bandwidth with better impedance matching and high antenna gain. The antenna was printed on a Roger RO4003 substrate with dielectric constant of 3.55, loss tangent of 0.002, and substrate thickness of 1.525 mm. The feeding network for the proposed 2 × 2 DLP antenna array is shown in Figure 3b with total array dimensions of Wsub × Lsub =120 × 130 mm^2^. It consists of 1-to-4 broad band Wilkinson power divider [30], extended from 1.8 GHz to 2.9 GHz. More details about the design are available in [5,6].

**Figure 4:**
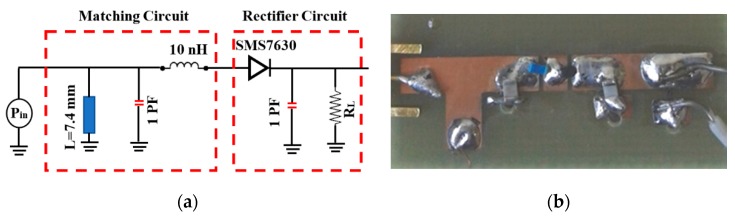
Multi-band rectifier circuit: (**a**) Schematic diagram; (**b**) Fabricated circuit.

A multi-band rectifier is used to rectify the received RF power at five frequencies of 1.8, 1.9, 2.1, 2.4, and 2.6 GHz. Figure 4a shows a schematic diagram of the proposed multiband rectifier circuit, while Figure 4b shows the fabricated circuit. The circuit consists of a matching circuit and a rectifier circuit. The matching circuit consists of short ended stub, 1 PF capacitor, and 10 nH inductor, to reduce the losses between the antenna and the rectifier for maximum power transfer. The rectifier circuit is a full wave rectifier, which uses a single Schottky SMS7630 diode, smoothing capacitor and load resistance of 10 KΩ. The SMS 7630 has a very low turn on voltage [39], which is suitable for the low values of received ambient power.

### 2.3. Power Management Unit Design

The power management unit (PMU) is considered as the bridge between the RF harvester and the IoT sensor node. The PMU is an essential block for boosting the output DC voltage from the harvester and generating the required voltage to power on the IoT sensor node. The proposed PMU cell is based on the commercial BQ25504 integrated circuit (IC). This IC is an ultra-low-power DC-to-DC converter with an embedded battery charger to save energy while the system is idle. It also has a maximum power point tracking (MPPT) circuit to ensure maximum power efficiency. In addition, it provides an adjustable output voltage range that varies from 2.2 V to 5.25 V [40]. Even though there are a lot of alternatives in the market, the BQ25504 has a very low quiescent current that equals 330 nA [40]. This makes it the most suitable solution for the proposed IoT system.

**Figure 5:**
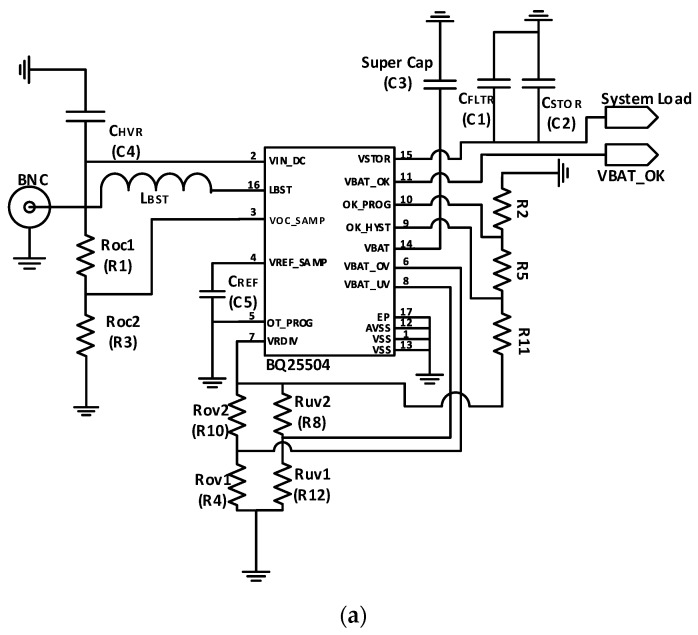

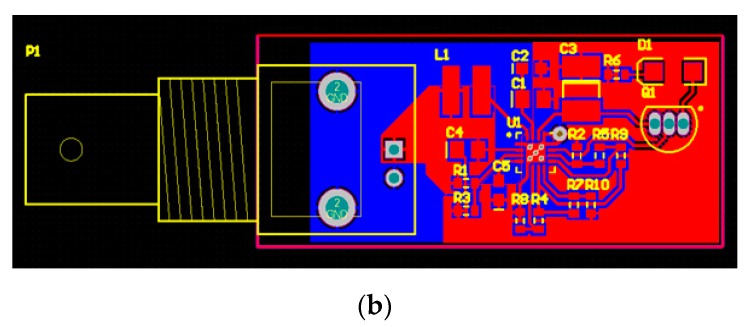
Power management unit (PMU): (**a**) Circuit schematic; (**b**) PCB Layout (41 × 19 mm^2^).

Figure 5a shows the circuit schematic of the PMU, where Roc1 and Roc2 are used to set the maximum power point (MPP) through a voltage divider. Rov1/Rov2 and Ruv1/Ruv2 are used to set the over-voltage and under-voltage thresholds, respectively [40]. In addition, a supercapacitor is utilized to provide the system with constant power. CFLTR/CSTOR are used to filter the high-frequency ripples [40]. Using the proper equations in [37], the charging voltage is set to be 3.7 V. The printed circuit board is shown in Figure 5b, where it occupies 41 mm × 19 mm.

### 2.4. IoT Architecture and System Design

The proposed IoT system design is based on a four-layer architecture driven from [41]. It has four layers: physical interface layer, network connectivity layer, fog processing layer, and cloud processing layer, as shown in Figure 1a.

#### 2.4.1. Physical Interface Layer

The physical interface layer is the lowest in the architecture. Its main role is to interface with the environment in two ways: by measuring the environment attributes (temperature, humidity, …, etc.) and by implementing any received actions from the upper layers (to control these environmental attributes by monitoring the operation of devices like HVACs and dehumidifiers). The physical layer interface is composed of two types of nodes:

**Sensor Node:** The sensor node collects measurements of the monitored museum environment attributes. These measurements are wirelessly transmitted through the network connectivity layer to the fog processing layer, where it is processed. Unlike related works [2,9,10,11,12], our sensor node is designed in a way that ensures minimum power consumption in order to be self-powered via the designed RF harvesting system.

The sensor nodes are built using the Texas Instruments multi-standard ultra-low power wireless microcontroller CC2650. The CC2650 has several features that make it suitable for energy harvesting applications. The device can be operated in a shutdown mode, consuming only 100 nA. In this mode, the microcontroller wakes up only on external interrupts. This feature is used with an external timer, a passive infrared (PIR) sensor, and an accelerometer to ensure the microcontroller is asleep, unless an event happens. The external timer is used to periodically wake up the microcontroller. The PIR sensor is used for occupancy detection and the accelerometer is used for touch detection. Environment attributes are measured using humidity, temperature, light, and CO and CO_2_ sensors. The components used in the prototype are tabulated in Table 1.

Another main part of the sensor node is the firmware of the microcontroller. It is very important and has a significant impact on the power consumption of the node. Therefore, the firmware is designed entirely with a focus on power-on times and power modes of the devices. The firmware is developed in an TI-RTOS environment, developed by Texas Instruments [42]. The TI-RTOS power manager module is used to push the microcontroller to the lowest power mode possible, at any time.

**Actuator Node:** The actuator node is the device that implements the decisions sent to it from the fog processing unit by controlling the devices that control the environment attributes in a museum section (air cooler, dehumidifier, …, etc.). The design of the actuator node is much simpler than the design of the sensor node, as it does not have the same power restrictions (it is powered through the mains). The main component of the actuator node is the microcontroller. The same controller used in the sensor node is used in the actuator node, the CC2650. This resolves most of the possible compatibility issues in the network connectivity layer.

#### 2.4.2. Network Connectivity Layer

The role of this layer is to connect the nodes in the physical interface layer with the fog processing layer. The ZigBee protocol is used to implement this role. ZigBee is a high-level communication protocol based on IEEE 802.15.4 personal area network standard [43]. It is designed mainly for low power applications that require low power consumption without interest in high data transfer rates. The ZigBee device used in the prototype is XBee ZigBee S2C, which has power-down current lower than 1 µA and operating current of 33 mA when transmitting data [44].

We consider a star topology for our ZigBee network, in which a single coordinator is implemented in the fog layer gateway. The coordinator’s indoor coverage is up to 90 m at a transmission rate of about 250 KB/sec [44]. Given that our packet size is 18 bytes, transmission delay is only 72 µsec.

#### 2.4.3. Fog-Processing Layer

The fog-processing layer’s main role is to control environmental attributes (temperature, humidity, light, CO and CO_2_) by sending commands to the actuator nodes. The actuator nodes then send the proper commands to the control devices (air cooler, dehumidifier, …, etc.). Even though the decisions could be made on cloud servers and sent directly to the nodes, they could be made on both the fog-processing unit or the cloud. This protects the system from being exposed to Internet connection issues (latency, drops and timeouts, …etc.). Some system features of the system do not accept high latencies and drops, such as the touch detection feature, which needs high responsiveness to instantaneously turn on the alarm when a theft is detected.

The fog-processing layer is implemented on a gateway node using Raspberry Pi board with the Raspbian operating system. This gateway node is the coordinator of the ZigBee network, enabling it to manage the ZigBee network, connect to all devices on the network, and change their operations wirelessly using the ZigBee protocol on-air update feature.

A programming tool called Node-Red is then used to build the application of the fog-processing layer. Node-Red is a programming tool that uses a visual wiring concept to connect hardware and cloud services with the APIs of the common applications used in IoT systems [45].

#### 2.4.4. Cloud-Processing Layer

The topmost layer in our system is the cloud-processing layer. This layer receives the sensors’ readings from the fog-processing layer and archives these readings in a database for further analysis. This layer gives museum managers the ability to monitor the museum’s ambience by accessing the database to see historical data or by accessing the received data directly in real-time. Finally, the results of deep-data analysis (discussed in the next section) are sent to the fog-processing unit to be used for better ambience control.

### 2.5. Deep Learning-based Time Series Prediction

**Figure 6:**
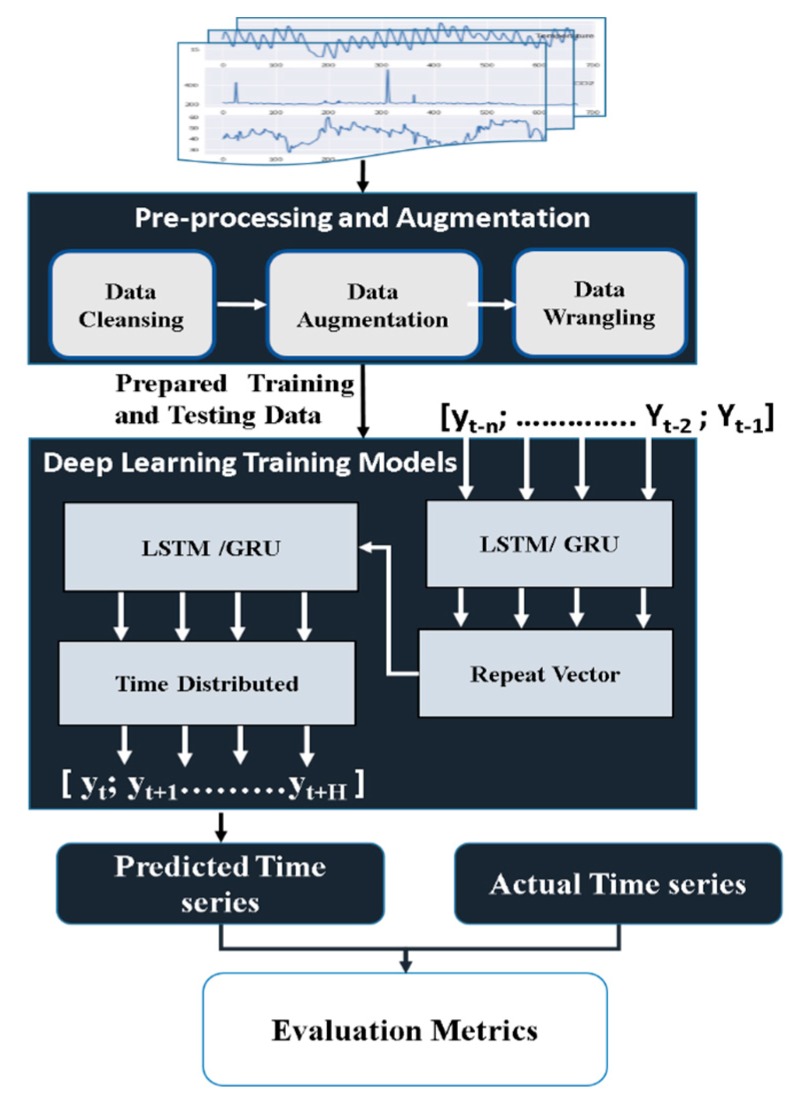
Deep learning-based time series prediction framework.

The IoT system performs instantaneous and time-critical actions based on the collected data. All the data is relayed to the cloud to be stored and further analyzed. In this section, we propose a deep learning-based time series prediction framework to reach optimized action plans based on the history of the collected data. The proposed deep learning models for multi-step ahead prediction is based on the Encoder-Decoder scheme, which aims to address sequence-to-sequence problems, and improves our preliminary design presented in [13]. Figure 6 illustrates the block diagram of the framework.

#### 2.5.1. Data Preprocessing and Augmentation

The goal of this phase is to prepare a raw time series and transform it to a proper representation for deep learning models through the following three steps: 

**Data Cleansing:** First, the time series data is cleaned by removing all duplicated values from the time series.

**Data Augmentation:** The size of the training dataset is then increased by generating a synthetic time series (using Average Selected with Distance (ASD) method [46]) to improve deep learning performance. Time series can be defined as an ordered set of real values and expressed as *S* = (*t_1_*, *t_2_*, …, *t_N_*), where *N* is the length of the time series. The main aim of our step is to take a set of time series belonging to the same day over the whole period in *S*, then, calculate a weighted average *W*, and use this average as a new synthetic time series. This synthetic time series is concatenated with the original one.

**Data Wrangling:** In this last step, the input data is first normalized to values in [0; 1]. The data is then divided into 100 and 20 days for training and testing datasets, respectively. Finally, the data is transformed to a supervised machine learning problem suitable to train the models in the form of (*F_t − n_, … F_t − 2_, F_t − 1_*) as input and (*F_t_, F_t + 1_, …, F_t + n_*) as output using a window of size 24 and a moving step of 1, where *n* equals 24.

#### 2.5.2. Prediction Phase

In this phase, LSTM [47] and the GRU [48] Networks based Encoder-Decoder scheme, which aims to address sequence-to-sequence problems, is proposed for multi-step ahead prediction. It enables the model to be used for supporting input sequences with variable length and predicting output sequences with variable length [49,50]. The problem of multi-step ahead time series prediction can be defined as predicting the next *H* values [*y_t_*; *y_t + 1_*; : : : ; *y_t+H_*] given the historical time series [*y_t − n_*; : : ; *y_t − 2_*; *y_t − 1_*], where *t* is the time unit.

The deep learning-based prediction phase works as follows. First, both the LSTM and GRU models are trained using training datasets. Then, the models are validated on testing datasets using Mean Absolute Error ($MAE=\frac{\sum_{i=1}^{N}\left|a_{i}-\;p_{i}\right|}{N}$) and Root Mean Square Error ($RMSE=\sqrt{\frac{1}{N}\sum_{i=1}^{N}{\left(a_{i}-\;p_{i}\right)}^{2}}$), where *a* is the actual value, *p* is the predicted value, and *N* is the total number of values.

## 3. Results and Discussion

**Figure 7:**
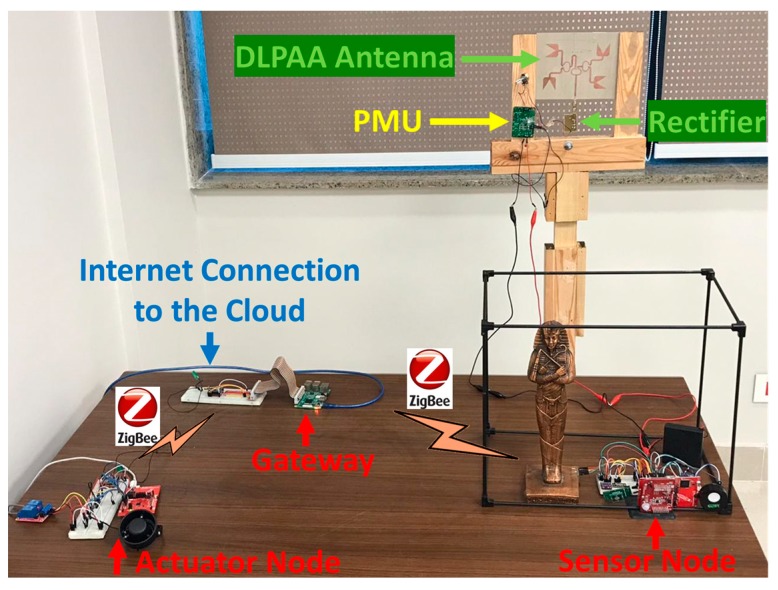
The developed prototype.

We build a prototype of the overall system that integrates all of its components, as shown in Figure 7. In this section, we present the experimental results that demonstrate the performance of the different components of the proposed museum control system.

### 3.1. RF Energy Harvesting Performance

**Figure 8:**
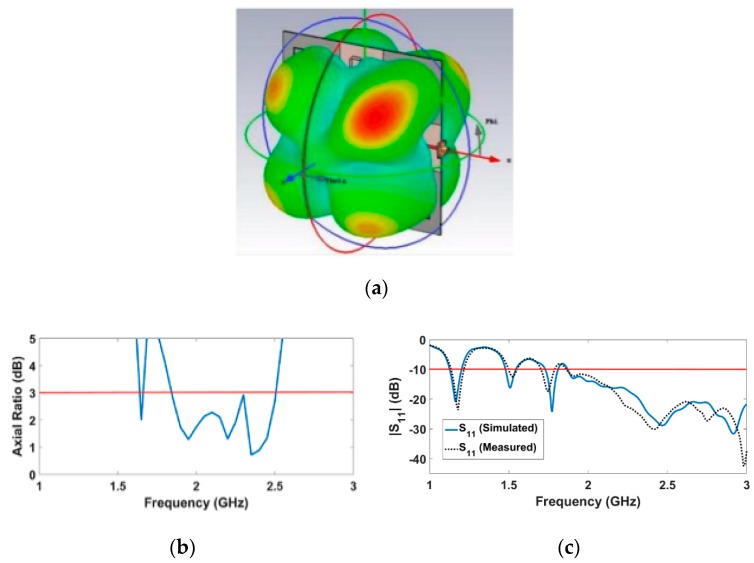
Characteristics of the 2 × 2 CP antenna array with sequential feeding at 2.45 GHz: (**a**) 3-D radiation pattern; (**b**) Achieved axial ratio; (**c**) Measured and simulated reflection coefficients [7].

The 3-D radiation pattern for the circularly polarized antenna array is shown in Figure 8. Four similar lobes can be noticed in the array pattern. Each element radiates according to its orientation and excited phase. The four lobes are directed towards +45°, +135°, –45°, and –135° with 30.5° angular width. Note that the single element has two lobes directed towards 0° and 180° with 87° angular width. Therefore, the array is more favorable in situations where the direction of the source is unknown and changing, as is the case with the considered scenario. In order to check the CP operation of the array, the axial ratio was calculated. The achieved axial ratio as a function of frequency is illustrated in Figure 8. This topology significantly enhances the axial ratio bandwidth (670 MHz) from 1.84 GHz to 2.51 GHz. A reflection coefficient of −28.39 dB was achieved at 2.45 GHz, as shown in Figure 8c.

**Figure 9:**
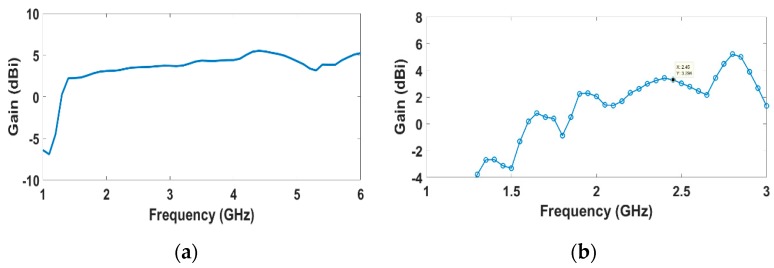
Achieved gain of the single element and 2 × 2 CP antenna array: (**a**) single antenna; (**b**) 2 × 2 array.

The gain, directivity, and radiation efficiency of the array are 3.29 dBi, 5.669 dBi, and 57.87%, respectively, at 2.45 GHz. The low radiation efficiency is attributed to the used substrate material, which is commercially available and has low cost at the expense of high dielectric loss. The gain variation of the 2 × 2 array is shown in Figure 9. It is worth noting that the total array gain is quite low, since each element is radiating in a specific direction. This case is desirable for ambient RF energy harvesting applications, since the positions of source and receiving antenna are relatively uncertain and consequently this array can collect signals from various directions simultaneously, when it is used as the receiving antenna in a rectenna structure.

Circular polarized rectennas are used due to their ability to obtain constant DC power at random polarization angles. However, to avoid 3dB CP loss, the DLPAA is used in our system, keeping the two main directions for maximum receiving power. Moreover, the Schottky SMS 7630 diode shows better performance with lower losses than HSMS 2860.

**Figure 10:**
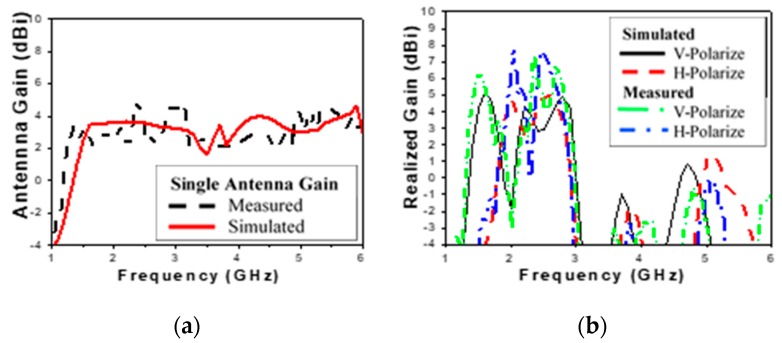
Simulated and measured horizontally and vertically polarized gain: (**a**) single antenna; (**b**) 2 × 2 array.

The horizontally polarized (H-pol) and vertically polarized (V-pol) gain of the DLPAA in a broadside direction are shown in Figure 10 The average gain is around 5.5 dBi for both V-pol and H-pol. The gain values of the antenna array at the frequencies correspond to the different wireless communication standards (GSM 1800, digital TV, Wi-Fi, and LTE) listed in Table 2. The average radiation efficiency of the designed DLPA array is about 95% over the operating band, as shown in Table 2. It was noticed that at different wireless communication frequencies, the H-pol and V-pol gains in the broadside direction are approximately the same.

**Figure 11:**
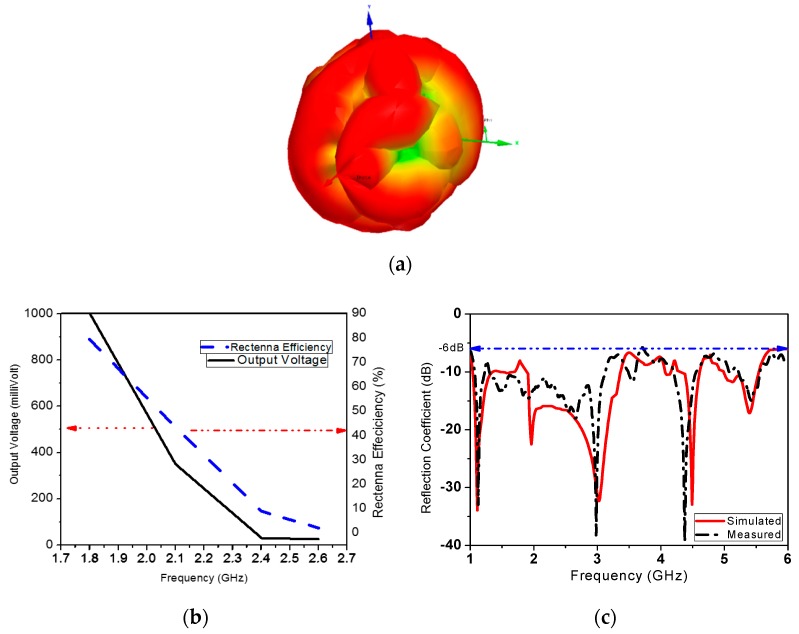
Characteristics of the 2 × 2 DLP array rectenna: (**a**) 3-D radiation pattern; (**b**) Output voltage and system efficiency; (**c**) Measured and simulated reflection coefficients [5].

To examine the received power of the designed single monopole element and DLPAA at certain frequencies, a DRG horn antenna (SAS-571) is used as a transmit antenna and an Anritsu MS27260 spectrum analyzer is used to measure the received power on the antenna element and the array for both vertical and horizontal polarizations. The input power of the transmit horn is 3 dBm two meters away from the receive antenna. Table 3 lists the received RF power in dBm for both the single element and antenna array. The received power for V-pol and H-pol of the antenna array are almost the same in the frequency bands of interest. The 3-D radiation pattern for the DLP array rectenna in all directions is shown in Figure 11a. The output voltage and efficiency of the system rectenna at different operating frequencies is shown in Figure 11b. The DLPAA achieves the maximum harvested voltage for the proposed system, which is 1000 mV, as shown in Figure 11b. It is achieved at 1.8 GHz with −9.1 dBm RF received power from a dedicated horn antenna source. A reflection coefficient of -18 dB has been achieved at 2.45 GHz, as shown in Figure 11c. 

Table 4 gives a comparison between the RF energy harvesting system proposed in this paper and the literature [51,52,53,54,55]. Table 4 shows that our proposed RF energy harvesting system has a larger number of covered frequency bands, reduced size, higher rectenna efficiency, higher RF sensitivity, and superior operation, as compared to different types of polarization techniques.

### 3.2. Power Management Unit Performance

**Figure 12:**
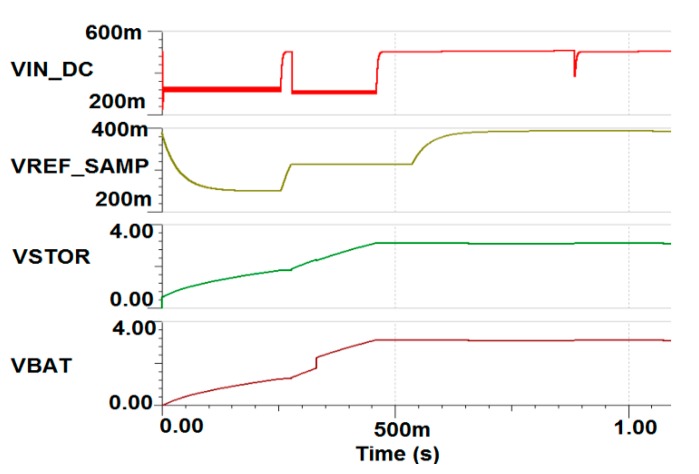
PMU simulation results.

The PMU circuit is simulated using TINA-TI, as shown in Figure 12, where transient analysis results show that the input voltage is kept less than 500 mV. The battery voltage (VBAT) reaches the typical value of 3.15 V after 400 msec, while the input current is limited to 400 µA.

**Figure 13:**
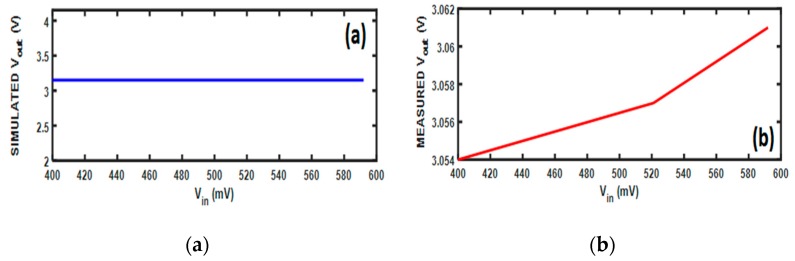
PMU simulation results versus the measurement results: (**a**) Simulation results; (**b**) Measurements results.

To test the PMU board, AA-3.7V-Lithium Ion 1200 mAh battery is charged using a DC voltage source with different values. The DC output voltage is expected to be constant around 3.15 V, as shown in Figure 13a. However, the measured results vary with a few millivolts around the required value, as shown in Figure 13b.

### 3.3. IoT System Performance

Here, we present the results obtained by implementation of a prototype using the IoT system design described in Section 4. First, we assess the lifetime of the node, and then present a set of experiments demonstrating the overall system performance.

The lifetime of the IoT system is limited by the lifetime of the sensor node, as it is the only battery-powered component in the system. To evaluate the lifetime of the sensor node, we measured power consumption in different power modes. The node has two power modes: sleep mode and active mode. In the sleep mode, the node consumes on average 2.5 mA. In the active mode, the node operates in three states, depending on which hardware elements and sensors are currently active. The current consumptions of the identified three active states are 50, 31.8, and 21 mA. Every time the node wakes up and enters the active mode, it takes only 0.5 second to collect sensor measurements and send them over the network to the gateway. The sensor node is powered by three of the used 1200 mAh LiFePO4 batteries with output voltage around 3.2 V to increase the current capacity to match the difference between the average RF energy harvesting rate and the high power consumption of the node when active. The lifetime of the node is 50.08 days, compared to 9.8 days in [11] and 20 hours in [2]. Table 5 presents a comparison of the proposed system with the closely related literature. It shows the extended lifetime of the system; despite the many functionalities it provides. 

Both antenna arrays (CP and DLPAA) are integrated with the sensor node through the PMU. The DLPAA array exhibits a charging rate of 1.8 mV/sec, which charges a 1F super-capacitor and 3.3 V 1600 mAh unloaded battery to 2 V in 19 min and 27 days, respectively. Meanwhile, the CP array shows a charging rate of 1.2 mV/sec, which charges a 1F super-capacitor and 3.3 V 1600 mAh unloaded battery to 2 V in 28 min and 39 days, respectively. Based on the power consumption of the sensor node, the proposed system is self-powered by charging its battery (or even using a super-capacitor) from the ambient RF energy, but not on the fly. This significantly extends the 50.08-day lifetime, when the node’s battery is continuously charged with the rates mentioned above. 

**Figure 14:**
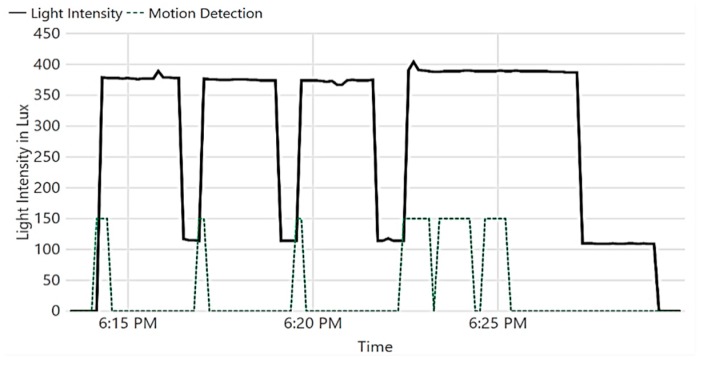
Automatic light control.

Next, we validate the IoT system’s capabilities to control the museum’s ambience. In the first experiment, we show how light is autonomously controlled based on the current visitor occupancy of a museum section. The results of the experiment are shown in Figure 14. At 6:14 pm, a visitor entered the section, causing light intensity to be changed to 380 Lux; he stood still for about two minutes. At 6:16 pm, the lights turned to dim (120 Lux), as no further movement was detected for 2 min (mean visitor standing time *T_mean_*). At 6:17 pm, when the visitor moved, the lights turned to 380 Lux again. Several visitors entered the area at 6:22 pm, causing consequent movement detections. When the visitors left the room at 6:25 pm, the lights were kept at 380 Lux for 2 min, then at 120 Lux for another 2 min, before being totally turned off. It is worth mentioning that *T_mean_* is configurable, and it is set to 2 min only for demonstration purposes.

**Figure 15:**
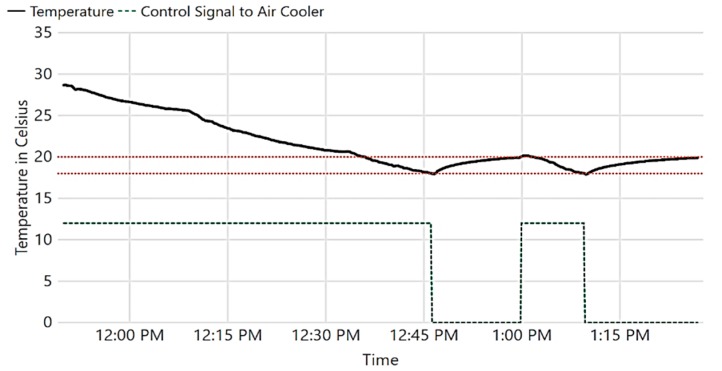
Automatic temperature control.

Another experiment was performed to demonstrate the IoT system’s ability to keep the temperature in the museum at a desired range, as shown in Figure 15. Note that rapid change in temperature is harmful to artifacts [1].

Initially, the temperature was intentionally increased, and the system turned on to monitor the temperature and control the operation of the air cooler. The air cooler was turned on until the temperature fell below 18 ℃ at 12:46 pm; the air cooler was then turned off, causing the temperature to increase. When the temperature passed 20 ℃ at 1:00 pm, the air cooler was turned on again.

### 3.4. Time Series Prediction Performance

We evaluate the performance of the proposed prediction framework using only readings of environment attributes that are measured more frequently, such as temperature, humidity, and CO_2_ sensors. Predicting the data of other sensors (PIR and touch detection) is not applicable, since these events are spontaneous. The proposed time series prediction system was trained and evaluated using the dataset collected by our sensor node. This dataset contains readings that are collected every one hour from temperature, humidity, and CO_2_ sensors over a period of 120 days. The data is then divided into 100 days for training and 20 days for testing. The collected data is uploaded into MySQL database on a cloud for more processing and prediction sequences. The reported experiments were conducted on Google Colab with K80 GPU and 12 GB memory. The proposed approach was designed with tensorflow and keras using a python environment on Linux platform. To validate the performance of the proposed framework for time series prediction, we calculated MAE and RMSE. Two models considering different input factors were trained. The first model input consists of target factor, day hour, and week. The second model’s input consists of humidity, temperature, CO_2_, day hour, and week day.

Table 6 presents the MAE and RMSE models of the predicted indoor CO_2_, temperature, and humidity on the testing dataset using original data. It illustrates that the best performance is obtained by the MAE (Model 1), considering the pattern of data for weekends, and working and non-working hours. Therefore, we will consider this model only with the augmented data, considering that date-time features reduce the RMSE for CO_2_ by more than 50% and for humidity by more than 10%, with slight enhancement in temperature RMSE, compared to [13].

Applying the data augmentation step generates 140 days of data; the generated data is then concatenated with the original one. Table 7 represents the MAE and RMSE of Model 1 for the predicted indoor CO_2_, temperature, and humidity on the testing dataset using augmented data. From the depicted experimental results in Table 6, it is concluded that using the data augmentation approach improves the performance of LSTM and GRU deep learning models by decreasing the RMSE by approximately 2.54% for CO_2_, 23.82% for temperature, and 2.59% for humidity, compared to [13].

**Figure 16:**
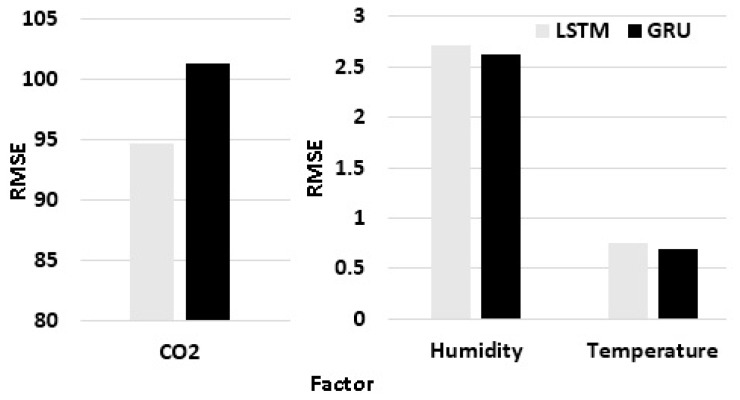
Prediction results using the augmented dataset.

Figure 16 shows that the LSTM deep learning model outperforms GRU for CO_2_, while the GRU model outperforms for temperature and humidity. This is due to the nature of the time series pattern for CO_2_, which is seriously affected by the number of occupants on weekends.

## 4. Conclusions

In this paper, we designed, implemented, and evaluated a low-power IoT system that uses RF energy harvesting to energize sensor nodes. More specifically, we presented the design and fabrication details of two different RF harvesting circuits—one using a circularly polarized antenna array and the other using a dual linearly polarized antenna array. A DLP array rectenna was used for the implemented prototype. The proposed array achieved 1000 mV and system efficiency of 79.5% at 1.8 GHz. The harvested energy is then boosted to the power level needed to charge the sensor node’s battery using a power management unit. The museum ambience data is communicated to the Internet for processing through a layered IoT architecture that was developed and implemented. Deep learning techniques were used to develop history-based predictions of museum ambience for the next 24 hours, based on which dynamic ambience control plans with low-energy consumption were created. Thereby, the museum control action plan combines both instantaneous sensor readings and foreseen environmental conditions to overcome faults in the sensors’ readings. Performance evaluation of the implemented prototypes does not only show the extended lifetime of the system but also its ability to effectively control museum ambience.

## Figures and Tables

**Table 1:** The components used in the prototype.

Component’s Part Number	Description
CC2650	Simple link multi-standard ultra-low power wireless microcontroller from Texas Instruments
TPL5110	Nano power system timer
HDC1010	Humidity sensor with a built-in temperature sensor
OPT3001	Ambient light sensor
ULPSM-CO 968-001	Ultra-low power analog sensor module for carbon monoxide
GC-0012	COZIR ultra-low power carbon dioxide sensor
LIS3DH	Ultra-low power 3-axis Nano accelerometer
BOOSTXL-TLV8544PIR	TLV8544 Quad Nano power Op amp PIR Motion Detector Demonstration Module

**Table 2:** Values of the realized gain in the broadside direction at different wireless frequency applications.

Freq. (GHz)	V-pol Gain (dBi)	H-pol Gain (dBi)	Rad. Efficiency (%)
1.8	3.5	1	85
2.1	1.5	5.3	95
2.4	6.3	5.4	94
2.5	5.3	7.5	99
2.6	4.5	6.7	98
2.8	5.3	4	99
2.9	3.2	1	92

**Table 3:** The ambient received power at different frequencies for the dual linearly polarized antenna array (DLPAA) in vertical and horizontal receiving modes (dBm).

Freq. (GHz)	Array Antenna	
	V	H
1.8	−34	−35
2.1	−36	−33
2.4	−34	−33
2.5	−35	−34
2.6	−36	−34
2.7	−35	−36
2.8	−36	−36
2.9	−38	−39

**Table 4:** Comparison between the proposed harvesting system and related technologies.

Work	Frequency (GHz)	Antenna Size (mm3)	Maximum Gain (dBi)	Rectenna Efficiency (%)	RF Sensitivity	Load(KΩ)	Polarization Type	Structure Simplicity	Technology
[51]	0.88–8.45	100 × 100 × 1.6	8.7	51.2% at 2.4 GHz	0 dBm	4.7	Linear	One layer	HSMS-2852
[52]	0.9	62 × 62 × 0.254	9	60% at 0.9 GHz	7 dBm	1	Linear	One layer	HSMS 2862
[53]	0.915 and 2.45	60 × 60 × 60	1.87 and 4.18	37% at 0.915 GHz	−9 dBm	2.2	Linear	One layer	SMS7630
[54]	0.915, 1.8 and 2.1	NA	NA	NA	−15 dBm	2.1	NA	NA	HSMS2850
[55]	0.9, 1.75, 2.15 and 2.45	155 × 155 × 1.52	9.8	59% at 0.9 GHz	-10 dBm	NA	Linear	Two stacked layers	SMS7630
Proposed	1.8, 2.1, 2.4, and 2.6	120 × 130 × 1.525	7.5	79.5% at 1.8 GHz	−15.5 dBm	10	Dual linear polarization and circular polarization	One layer	SMS7630

**Table 5:** Comparative Study of the Proposed IoT System to Closely-Related Systems.

	Architecture	Functionalities	Sensors	Microcontroller	Wireless Transceiver	Current in Sleep	Energy Harvesting	Lifetime Estimate
This paper	4-Layer IoT Architecture	Environmental monitoring & control Occupancy detection Touch detection	T ^1^, H ^2^, L ^3^, CO_2_, CO, Acc ^4^, PIR ^5^	Texas Instruments CC2650	XBee Pro X2C	2.5 mA	Yes	50.08 days
[2]	Adhoc Hopping	Environmental monitoring	T, H, L	PIC24F16KA102	nRF24L01	N/A	No	20 hours
[11]	3-Layer IoT Architecture	Environmental monitoring & control, Occupancy detection, Touch detection	T, H, L, Acc, PIR	Espressif ESP32	Embedded transceiver	5.09 mA	No	9.8 days
[12]	Customized	Environmental monitoring	T, H, L, Acc, Bar ^6^	MTS400CA	Embedded transceiver	N/A	No	A few days

^1^ T = Temperature, ^2^ H = Humidity, ^3^ L = Light, ^4^ Acc = Accelerometer, ^5^ PIR = Passive InfraRed, ^6^ Bar = Barometric Pressure.

**Table 6:** Statistical error parameters for LSTM and GRU deep learning models for indoor air quality using original data.

Target Factor	Model	Performance Metric			
		LSTM		GRU	
		MAE	RMSE	MAE	RMSE
*CO_2_*	*Model 1*	60.722	97.243	65.206	102.589
	*Model 2*	73.353	118.201	67.252	106.115
*Humidity*	*Model 1*	2.123	2.780	1.930	2.603
	*Model 2*	2.522	3.394	2.255	3.052
*Temperature*	*Model 1*	0.683	0.847	0.722	0.911
	*Model 2*	0.737	0.957	0.799	1.019

**Table 7:** Statistical error parameters for LSTM and GRU deep learning models for indoor air quality using augmented data.

Indoor Air Quality Factor	Performance Metric			
	LSTM		GRU	
	MAE	RMSE	MAE	RMSE
*CO_2_*	63.153	94.771	59.257	101.31
*Humidity*	1.893	2.708	1.861	2.630
*Temperature*	0.596	0.757	0.547	0.694
